# Basal Narcosis
*A Bristol University Post-graduate Lecture.


**Published:** 1939

**Authors:** E. G. Bradbeer

**Affiliations:** Honorary Anæsthetist, Bristol Royal Infirmary; Lecturer in Charge of Department of Anæsthesia, Universtiy of Bristol


### BASAL NARCOSIS.*

BY

E. G. Bradbeer, M.R.C.S., L.R.C.P., D.A.,

*Honorary Anæsthetist, Bristol Royal Infirmary; Lecturer in Charge of Department of Anæsthesia, University of Bristol.*

---

Basal or pre-anæsthetic narcosis is the procedure by which we are able to modify or eliminate the immediate pre-operative apprehension felt by the patient during his transport to the anæsthetic room, and the fear of the actual induction of anæsthesia. It also permits the choice of weaker and less poisonous anæsthetic agents for the maintenance of adequate anæsthesia.

The possibilities of this procedure were first visualized in 1869 by a French physician, Claude Bernard, who was the first to give a hypodermic injection of morphine as a basal narcotic before administering ether or chloroform. As with the methods of anæsthesia itself, however, very little further real progress was made until the last twenty or thirty years. During the past ten years it has become the exception to administer an anæsthetic without some form of basal narcosis, except where circumstances contra-indicate. The chief contra-indications are (1) insufficient staff for adequate supervision of a large number of operation cases during a lengthened recovery-period, and (2) the condition of the patient.

* A Bristol University Post-graduate Lecture.

### RISKS.

At the outset I would like to give a word of warning. Let us not in every case adopt this procedure and try to secure complete oblivion before commencing the anæsthetic proper, without carefully considering any possible risk to the patient's health, and indeed to life itself. Do not lose sight of the primary object of the method, which is to eliminate apprehension, and so to reduce psychological shock. It may be very spectacular to render every patient completely unconscious before leaving the ward, but in order to do this we must administer full doses of the drugs, and in doing so we may find ourselves confronted with risks which place the patient in a precarious position.

These risks fall into two categories. (*a*) Where the patient's reaction to the drug is normal. (*b*) Where the patient has an idiosyncrasy to the drug.

*Normal risks.*

1. Obstruction of airway. Respiratory depression is a prominent feature of most of the basal narcotics, and this, in combination with the possibility of relaxation of the jaw carrying the back of the tongue on to the posterior pharyngeal wall, may produce a partial or complete asphyxia without much visible respiratory effort on the part of the patient. So that it is of vital importance that a skilled nurse should be in uninterrupted attendance until the patient has recovered consciousness. The deeper the basal narcosis, and the more toxic and longer-acting the general anæsthetic chosen, the longer will be the post-operative period during which the patient is subject to this hazard. The minimum amount of narcotic should be used to effect the required result, followed by anæsthesia by one of the quickly eliminated, non-toxic gaseous anæsthetics.

2. Reduced pulmonary ventilation. Owing to the respiratory depression, deficient aeration of the lungs, particularly if of long duration, may lead to pulmonary complications.

3. The drug is irretrievable. The narcosis, which may have been satisfactory before commencement of the anæsthetic and the operation, may become dangerously deep if shock develops during the operation, or if there has been undue hæmorrhage. The anæsthetic may be discontinued, but not so the basal narcotic.

4. Liver damage. Drugs of the barbiturate group and avertin are detoxicated in the liver, and are unsuitable in cases in which there is deficient liver function.

5. Injury. Occasionally, during the recovery period, no matter what the narcotic used, there may be violent restlessness, during which the patient may cause himself some injury, *e.g.* through falling out of bed. Here again we must be able to rely fully on a competent nurse to watch the patient, and to administer a suitable sedative if necessary. Another risk is that the patient may waken and appear quite rational, but actually may be in a state of amnesia and not responsible for his actions, and will still require observation.

One case is reported in which the patient awoke in this condition and asked for a cigarette; the nurse, satisfied that he was awake, allowed him to light a cigarette and then left the room. On looking into the room shortly after, she found the patient fast asleep and the bed-tablecloth ablaze. The patient subsequently had no recollection of either asking for or lighting a cigarette.

Another case occurred in my own experience some years ago, in which I administered pernocton to a lady before an operation for abdominal hysterectomy. The anæsthetic was administered and the operation performed without incident. When she awoke about three hours later, she held intelligent conversation with the nurse, who was satisfied that she was awake and so left the room. On looking in five minutes later she found the patient out of bed and dressing to go home. She was put back to bed, and fortunately no complications arose. Here again on the following day the patient had not the slightest remembrance of her escapade, and had thoroughly enjoyed her anæsthetic.

*Idiosyncrasy.*—Some patients are unduly susceptible to certain drugs, so that average doses produce deep narcosis, and all the dangers in the first category are exaggerated. If the more toxic drugs are being used, they should not be administered in full doses until the response to a modified dose has been observed. It has been noted that allergic patients are sometimes found to be unduly sensitive to the narcotic drugs.

### ADVANTAGES.

The advantages of basal narcosis are :—

1. Psychic shock reduced.

2. Subsequent anæsthesia may be maintained with gas and oxygen alone. If it is necessary to use ether or chloroform for further relaxation, small amounts only are necessary. So that ether, instead of being the main anæsthetic, is an accessory.

3. Immediate after-pain is lessened.

4. Post-operative vomiting is less frequent, owing largely to the smaller amount of ether or chloroform used.

### ADMINISTRATION.

I believe that in the majority of cases mild hypnosis is all that is needed, that is, for the patient to arrive in the anæsthetic room sufficiently drugged to be in that happy state of not really caring what happens next. Actually in a large proportion of cases, where a moderate dose of the drug is administered, the patient may be apparently awake and yet is completely amnesic and has no memory of leaving the ward. However, each patient must be seen by the anæsthetist before the day of operation where possible and the drug and dosage in each case must be adjusted according to temperament, general condition, and the anæsthetic to be subsequently used.

*Temperament.*—A small percentage of patients prefer not to have basal narcosis. In these cases it is as well to withold it unless it is considered to be essential to reduce the amount of inhalation anæsthetic to a minimum. A large number like to have basal narcosis, but do not mind if they are not completely unconscious. A considerable percentage express the wish that they would definitely like to know nothing after leaving bed. The members of a small group get into a panicky condition of mind, and with these one feels that one must be certain that they are quite unconscious before they leave their beds.

*General condition.*—Elderly patients, and all those suffering from a considerable degree of toxæmia or cachexia, should receive a dose below the average. Toxic goitre provides an exception to this rule and calls for a dose above the average.

*The anæsthetic to be subsequently used.*—The gaseous anæsthetics are preferable after all forms of basal narcosis. Vinyl ether, ether or chloroform may be used as supplements if further relaxation is required.

As a general rule a darkened room and quietness are conducive to successful basal narcosis, but in my opinion the patient should not be left once the drug has been administered, and this constant attendance should be continued until the patient has returned to full consciousness. The drugs at our disposal are administered hypodermically, intravenously, orally or rectally according to their characteristics. Any dose which one may mention is merely an indication of a moderate dose, which it is wise not to exceed until the anæsthetist has had the opportunity over a series of cases to observe its effect on the induction and maintenance of anæsthesia and the subsequent condition of the patient.

*Morphine* gr. $\frac{1}{6}$ with *Hyoscine* gr. $\frac{1}{100}$ produce amnesia, and are useful in cases where the nature of the operation precludes the use of a rectal injection. They are best given three-quarters to one hour before the operation. They tend to produce respiratory depression and often render induction with ether or chloroform more difficult. It is wise to avoid the use of morphine for children.

*Paraldehyde.*—This is the safest basal narcotic, and is administered rectally in saline, tap water or olive oil. The advantage of giving it in olive oil is that the free solubility in oil allows the total dose to be given in small bulk, but as absorption is much slower it has to be given two hours before operation. There is no respiratory depression, and the cough reflex is not abolished. It has no deleterious effect on the liver or kidneys. Given in a dosage of $\frac{3}{4}$ drachm per stone (maximum dose 8 drachms), it is equally useful for adults or children. The general precautions as to supervision, however, are absolutely essential. Restlessness during the recovery period is readily controlled by a small dose of opiate. If given in tap-water or saline (paraldehyde 1 drachm in saline 1 ounce), it is best administered one and a quarter hours before operation. The patient should lie on his left side during the injection, and greater success will be obtained by giving it very slowly. To avoid tragic misunderstandings, it is wise to avoid ordering basal narcotics by telephone, and invariably to order in writing, using English figures and the written measure, *e.g.* "6 drachms."

*Barbiturates.*—A large number of proprietary preparations of this group are in use for the purpose of basal narcosis. Some are given orally, some intravenously and some per rectum. They are all respiratory depressants, are detoxicated by the liver and excreted by the kidneys. Liver malfunction is a contra-indication.

*Nembutal.*—This may be given intravenously, orally or rectally. It is generally administered by the mouth one hour before operation. A moderate adult dose would be 3 grains, and this is sometimes combined with morphine gr. $\frac{1}{6}$ hypodermically. If used intravenously, a freshly prepared 5 per cent. solution in distilled water is injected at the rate of 1 c.cm. per minute. The patient is instructed to count aloud, and when he ceases to count the injection is stopped. Dosage in this manner is more accurate than by the oral route. Sodium amytal is used in a similar manner.

*Pernocton* is only administered intravenously as described for nembutal. It is stable in solution and so mixing and dilution is unnecessary.

*Evipan* is an anæsthetic in itself, but is very useful as a basal narcotic. The solution must be freshly prepared and is injected at the rate of 3 to 4 c.cm. per minute; when the patient has ceased to count, the amount already given is noted and as much again is injected at the same rate. The advantage of evipan is the rapidity of its elimination, so that reflexes return as soon as the subsequent anæsthetic is discontinued.

These intravenous barbiturates are particularly useful in the group of cases indicated previously, where one has to guarantee the patient will be asleep before leaving his bed. There are a number of other barbiturates in use. It is advisable that subsequent anæsthesia should be maintained as far as possible by the gaseous anæsthetics.

*Avertin*—is used by rectal injection, administered half an hour before operation. Narcosis is perhaps a little more certain than with paraldehyde and there is no smell. Strict attention must be paid to the instructions on the chart supplied, as regards dosage, mixing and testing the fluid. It is more toxic than paraldehyde, and is contra-indicated in liver disease. It is particularly useful for thyroidectomy.

